# Maternal human telomerase reverse transcriptase variants are associated with preterm labor and preterm premature rupture of membranes

**DOI:** 10.1371/journal.pone.0195963

**Published:** 2018-05-17

**Authors:** Caroline Marrs, Kevin Chesmore, Ramkumar Menon, Scott Williams

**Affiliations:** 1 The University of Texas Medical Branch, Division of Maternal-Fetal Medicine, Galveston TX, United States of America; 2 Geisel School of Medicine, Dartmouth College, Hanover NH, United States of America; 3 Case Western Reserve University, Cleveland OH, United States of America; John Hunter Hospital, AUSTRALIA

## Abstract

**Objective:**

Premature aging and short telomere lengths of fetal tissues are associated with spontaneous preterm labor (PTL) and preterm premature rupture of membranes (pPROM). Maintenance of telomere length is performed by the enzyme telomerase. Human telomerase reverse transcriptase (hTERT) is a subunit of telomerase, and its dysfunction affects telomere shortening. This study assessed whether maternal or fetal genetic variations in the hTERT gene are associated with PTL or pPROM.

**Methods:**

A case (PTL or pPROM) control (term birth) genetic association study was conducted in 654 non-Hispanic white mothers (438 term, 162 PTL, 54 pPROM) and 502 non-Hispanic white newborns (346 term, 116 PTB, 40 pPROM). Maternal and fetal DNA samples were genotyped for 23 single nucleotide polymorphisms (SNPs) within the hTERT gene. Allele frequencies were compared between cases and controls, stratified by PTL and pPROM. Maternal and fetal data were analyzed separately.

**Results:**

Allelic differences in one SNP of hTERT (rs2853690) were significantly associated with both PTL (adjusted OR 2.24, 95%CI 1.64–3.06, p = 2.32e-05) and with pPROM (adjusted OR 7.54, 95%CI 3.96–14.33, p = 2.39e-07) in maternal DNA. There was no significant association between the hTERT SNPs analyzed and PTL or pPROM in the fetal samples.

**Conclusion:**

hTERT polymorphisms in fetal DNA do not associate with PTL or pPROM risk; however, maternal genetic variations in hTERT may play a contributory role in risk of PTL and PPROM.

## Background

Preterm birth (PTB) is the leading cause of neonatal morbidity and mortality.[1] It complicates 5–12% of pregnancies[2], and infant sequelae include respiratory distress syndrome, necrotizing enterocolitis, intraventricular hemorrhage, and sepsis, among many others. While some preterm births are medically indicated for either maternal or fetal benefit, others are spontaneous (preterm labor [PTL] or preterm premature rupture of membranes [pPROM]). The precise biological signals and mechanisms that determine human parturition, term or preterm, continue to be poorly understood. There are likely multiple and redundant pathways that converge on the phenotype of preterm and term parturition.[3] One of the mechanisms initiating parturition that our laboratory has reported recently is fetal membrane cell senescence. The core hypothesis is that fetal amnion and chorion senescence and senescence-associated inflammation is the initiator of a coordinated cascade leading to parturition.[4]

Evidence for fetal membrane senescence has been accumulating. In the second half of pregnancy, there is a gestational age-dependent reduction in telomere length of fetal leukocytes, which has been shown to strongly correlate with telomere length of fetal membrane cells.[5] At term, labor is associated with accelerated telomere shortening in fetal membrane tissues.[6] It is thought that the oxidative stress and inflammation of labor contributes to telomere attrition. Behnia et al reported increases in several markers of senescence from pregnancies in labor compared to those sampled prior to the onset of labor.[7] To establish causality, the same group established an in-vitro model of fetal membrane organ explant cultures and primary amnion epithelial cells from term not-in-labor tissue that showed oxidative stress induces acceleration of telomere shortening and senescence and produces significant inflammation.[6]

Replicative senescence is a telomere-dependent aging process that associates with a reduced risk of cancer.[8,9] Telomeres are guanine rich caps on the ends of chromosomes that function to protect chromosome integrity. Telomeres shorten over time due to incomplete replication with each cell cycle, and therefore telomere length is one marker of cell aging.[10,11] Telomere dysfunction is thought to be one of many signals that can induce premature cell senescence.[9,11] Telomerase is the enzyme that maintains telomere length (thereby postponing cell-cycle arrest) by adding nucleoside repeat sequences to the 3 prime end of DNA.[12] It is made of two components: the human RNA subunit that acts as the template and the human telomerase reverse transcriptase (hTERT), that adds the nucleosides.[10]

There is a robust literature describing hTERT polymorphisms associated with phenotypes of both slowed aging, such as cancer,[13–16] as well as accelerated aging, such as cardiovascular diseases.[17–21] Specifically, shortened telomere length has been shown to be associated with carotid atherosclerosis,[17] stroke, myocardial infarction, type 2 diabetes,[18] and coronary artery disease.[19,22] Haycock et al recently published a large genome wide association study (GWAS) that showed a protective effect of genetically increased telomere length on the development of coronary heart disease and abdominal aortic aneurysm, as well as other non-cardiovascular diseases.[22]

It is thought that the association of hTERT polymorphisms and clinical disease is mediated through changes in telomere length. A few studies have shown associations between changes in telomere length and genetic variants of hTERT,[15,23] as well as other genes.[24] These studies suggest that, in addition to aging, genetic factors influence telomere length.

Fetal tissue telomere length differences are also associated with adverse pregnancy outcomes. In pregnancies affected by pPROM, fetal leukocytes had significantly shorter telomeres, compared to gestational age matched samples from PTL.[5] The telomere lengths are also smaller than those from the term birth samples. This suggests that dysfunctional or accelerated aging of fetal membranes, evidenced by shorter telomeres, may contribute to pPROM. It also may indicate that the aging mechanism leading to pPROM differs from that leading to PTL with intact membranes.[25]

To summarize, normal aging of fetal cells is a physiologic process linked to term parturition. However, accelerated telomere shortening is linked to premature aging and adverse pregnancy outcomes, including pPROM. It has been hypothesized that telomere shortening acts as a biologic clock, affecting the timing of labor.[4] Because telomerase regulates telomere length, perhaps the accelerated telomere shortening seen in pPROM is related to reduced fetal telomerase activity. It has also been suggested that maternal decidual senescence plays a role in the mechanisms of parturition,[26] and therefore maternal telomerase activity may contribute to the phenotype of spontaneous preterm birth, as well.

There is ample evidence that genetics plays a role in the phenotype of preterm birth.[27–29] While hTERT polymorphisms have been shown to be associated with certain types of cancers,[13–15] and other diseases,[17–19,22] there have been no genetic association studies investigating the association of hTERT polymorphisms and PTL or pPROM. hTERT has a biologically plausible role in spontaneous preterm birth via the fetal cell senescence pathway.

Our data thus far suggests that the two phenotypes of spontaneous PTL and pPROM are due to different aging mechanisms, maybe even in different tissues. The purpose of this study was to determine if maternal or fetal genetic variations (single nucleotide polymorphisms [SNPs]) in the hTERT gene are associated with PTL or pPROM.

## Materials and methods

### Study design

Institutional review boards at TriStar Nashville, TN and The University of Texas Medical Branch at Galveston, TX, approved this study. Subjects provided informed written consent to use their biological specimens for various studies related to preterm birth. Genotype and covariate data can be found in the Supplement (S1 Table).

We performed a case control genetic association study in women and newborns with PTL or pPROM compared to those with term birth. This study utilized previously banked samples from the Nashville Birth Cohort, which recruited women from the Centennial Medical Center in Nashville, TN between September 2003 and December 2006. The methods have been previously reported,[28] but briefly, the original cohort inclusion criteria were women aged 18–40 years with a singleton, live birth. Women were excluded if their pregnancy was complicated by multiple gestation, preeclampsia, placenta previa, fetal anomalies, gestational diabetes, polyhydramnios, oligohydramnios, or surgery. Our analyses further excluded women or newborns with missing genotypes. Racial disparity in genetic predisposition to both preterm birth[29] and telomere length[30–31] has been reported previously; in addition, our biobank had low numbers of remaining non-Caucasian samples. Hence, our study was restricted to the Caucasian population.

Cases of PTL were defined as delivery at less than 36 0/7 weeks gestation preceded by preterm labor with intact membranes. Cases of pPROM were defined as delivery at less than 36 0/7 weeks gestation preceded by premature rupture of membranes. A cut-off of 36 0/7 weeks gestation was used to correct for the lack of precision of pregnancy dating and to ensure a truly different phenotype than those with term birth. Controls were women or newborns with term birth, defined as 37 0/7 weeks of gestation or more.

### Methods

Demographic and clinical data were obtained from questionnaires and medical records. Gestational age was determined by last menstrual period and corroborated by ultrasound dating. Race was determined by self-report and a questionnaire that traces ethnicity back two generations from the parents.

Maternal blood was collected at the time of admission for delivery either at term or preterm, and cord blood was collected at delivery. DNA was isolated from maternal and neonatal cord blood using the Autopure automated system (Gentra Systems, Minnesota, MN). 23 SNPs from the hTERT gene were genotyped using the Sequenom platform (Table 1). Those SNPs that genotyped with low efficiency on Sequenom were re-genotyped using Taqman assays; this occurred with one SNP: rs2736100. We chose tagSNPs in low linkage disequilibrium using the website https://snpinfo.niehs.nih.gov/. For quality control, SNPs were not analyzed if they deviated from the Hardy-Weinberg equilibrium (p<0.001), had a low minor allele frequency (<0.05), or had low genotyping efficiency (<0.95).

**Table 1:** hTERT SNPs that were genotyped for maternal and fetal analyses and those that were excluded from analysis.

hTERT SNP	Included in maternal analyses?	Reason for exclusion from maternal analyses	Included in fetal analyses?	Reason for exclusion from fetal analyses
rs2736114	Y	NA	Y	NA
rs2075786	Y	NA	Y	NA
rs4246742	Y	NA	Y	NA
rs4975605	Y	NA	Y	NA
rs10069690	Y	NA	Y	NA
rs2242652	Y	NA	Y	NA
rs2853677	Y	NA	Y	NA
rs2853672	Y	NA	Y	NA
rs2853690	Y	NA	N	Low GE
rs2853676	Y	NA	N	Deviated from HWE
rs2736098	N	Low GE	N	Low GE
rs121918664	N	Low MAF	N	Low MAF
rs35719940	N	Low MAF	N	Low MAF
rs387907251	N	Low MAF	N	Low MAF
rs121918666	N	Low MAF	N	Low MAF
rs199422301	N	Low MAF	N	Low MAF
rs121918663	N	Low MAF	N	Low MAF
rs387907249	N	Low MAF	N	Low MAF
rs199422297	N	Low MAF	N	Low MAF
rs199422294	N	Low MAF	N	Low MAF
rs34094720	N	Low MAF	N	Low MAF
rs121918661	N	Low MAF	N	Low MAF
rs387907247	N	Low MAF	N	Low MAF

SNP: single nucleotide polymorphism, GE: genotyping efficiency, MAF: minor allele frequency, HWE: Hardy-Weinberg equilibrium

### Statistical analysis

We performed 4 separate case-control analyses as PTL and pPROM were analyzed separately and maternal and fetal DNA were also analyzed separately. All analyses assumed an additive genetic model.

Maternal demographic and maternal and neonatal outcome data were compared between cases and controls using student’s t, Chi-squared, or Mann-Whitney U tests, where appropriate. These tests were performed using R software. Allele frequencies for each SNP were compared between cases and controls using Chi-squared test. We then performed logistic regression analysis (PLINK v1.07) to determine the association of each SNP with the outcome of interest, adjusting for baseline variables that are known risk factors for the outcome of interest, or differed on simple comparison between cases and controls with a p value of less than 0.05. For each regression analysis, p-values thresholds were chosen using the Bonferroni correction for multiple comparisons.

## Results

The original cohort had 3,496 participants, including both maternal and fetal samples. In our nested case-control study, for the maternal univariate analyses, there were 162 cases of PTL, 54 cases of pPROM, and 438 controls. For the fetal univariate analyses, there were 116 cases of PTL, 40 cases of pPROM, and 346 controls.

### SNP filtering

Of the 23 SNPs chosen for our study, 13 were excluded from the maternal analyses (12 for low minor allele frequency and 1 for low genotyping efficiency), leaving 10 SNPs for analysis. In the fetal analyses, 15 SNPs were excluded (12 for low minor allele frequency, 2 for low genotyping efficiency, and 1 for deviation from the Hardy Weinberg equilibrium), leaving 8 for analysis (Table 1). The average genotyping rate in the remaining SNPs was 0.99.

### Baseline characteristics

Maternal and newborn baseline characteristics are described in Tables 2 and 3. As expected, significant differences between cases and controls were observed for gestational age, birth weight, and several measures of socioeconomic status. APGAR scores were better for controls, also as expected. A detailed description of the distribution of APGAR scores can be found in the Supplement (S2 and S3 Tables).

**Table 2:** Baseline characteristics of maternal cases and controls.

		Maternal Controls N = 438	Maternal PTL Cases N = 162	P value	Maternal pPROM Cases N = 54	P value
Maternal age (y)		28.4 ± 5.8	27.6 ± 5.8	0.13	25.8 ± 5.7	0.004
Nulliparity		31% (134)	30% (48)	0.90	39% (21)	0.28
Weight (pounds)		153 ± 37	164 ± 48	0.02	149 ± 47	0.004
Education				<0.001		<0.001
	<12 years	58% (252)	93% (150)		96% (52)	
	≥ 12 years	42% (186)	7% (12)		4% (2)	
Income				<0.001		<0.001
	<15K	18% (79)	33% (53)		48% (26)	
	15–25K	18% (79)	11% (18)		4% (2)	
	25–50K	25% (111)	36% (59)		33% (18)	
	50–100K	26% (116)	18% (29)		13% (7)	
	>100K	9% (40)	2% (3)		0% (0)	
Marital Status				0.38		<0.001
	Unmarried	23% (102)	29% (47)		48% (26)	
	Married	72% (315)	67% (109)		50% (27)	
	Not recorded	3% (12)	2% (4)		0% (0)	
Insured		78% (342)	93% (151)	<0.001	96% (52)	0.003
Smoker		12% (52)	19% (31)	0.03	16% (10)	0.23
Birth weight (grams)		3376 ± 526	2420 ± 824	<0.001	2288 ± 1074	<0.001
Neonatal female sex		46% (200)	43% (69)	0.99	48% (26)	0.04

PTL: preterm labor, pPROM: preterm premature rupture of membranes

**Table 3:** Baseline characteristics of fetal cases and controls.

		Controls N = 438	Infant PTL Cases N = 162	P value	Infant pPROM Cases N = 40	P value
Maternal age (y)		28.3 ± 5.9	28.4 ± 5.7	0.88	26.6 ± 6.4	0.11
Nulliparity		24% (107)	19% (31)	0.50	30% (12)	1.0
Weight (pounds)		154 ± 38	161 ± 46	0.17	155 ± 43	0.97
Education				<0.001		<0.001
	<12 years	45% (197)	65% (105)		93% (37)	
	≥ 12 years	34% (150	6% (10)		7% (3)	
Income				0.03		0.003
	<15K	15% (64)	19% (30)		40% (16)	
	15–25K	16% (68)	12% (19)		18% (7)	
	25–50K	20% (86)	25% (40)		28% (11)	
	50–100K	21% (92)	14% (22)		10% (4)	
	>100K	6% (28)	2% (4)		0% (0)	
Marital Status				0.45		0.003
	Unmarried	20% (86)	22% (35)		50% (20)	
	Married	55% (243)	45% (73)		45% (18)	
Insured		61% (269)	64% (104)	0.004	93% (37)	0.05
Smoker		10% (45)	15% (24)	0.05	28% (11)	0.03
Birth weight (grams)		3353 ± 506	2611 ± 768	<0.001	2640 ± 969	<0.001
Neonatal female sex		38% (165)	31% (51)	0.80	63% (25)	0.03

PTL: preterm labor, pPROM: preterm premature rupture of membranes

### Maternal SNP and PTL association

The maternal SNP minor allele frequencies among 162 PTL cases and 438 controls and the results of univariate analysis may be found in the supplement (S4 Table). For the maternal analyses, using the Bonferroni correction for multiple comparisons, the threshold for significance was set at p <0.005.

Of the 10 maternal SNPs analyzed, one (rs2853690) was associated with PTL in the unadjusted model (OR 2.42, 95%CI 1.93–3.05, p = 8.10e-11) (S4 Table). Logistic regression analysis was also performed to assess the association with PTL after adjusting for possible confounders (Table 4). The model adjusted for maternal weight, education (graduate high school or not), income (annual <$15,000, $15,000–24,999, $25,000–49,999, $50,000–99,999, or ≥$100,000), smoking status (Y/N), and insurance status (Y/N). 105 cases and 389 controls were included in the logistic regression, after removing 57 cases and 49 controls with missing covariate data. The SNP, rs2853690, remained significant in the adjusted model (aOR 2.24, 95% CI 1.64–3.06, p = 2.32e-05). No other SNPs were significant after multiple testing correction in either the adjusted or the unadjusted models.

**Table 4:** Logistic regression results for maternal single locus allele frequencies among cases and controls and association with preterm labor.

SNP	Minor allele	MAF Term	MAF PTL	aOR (95% CI)	P value
rs2853690	A	0.26	0.46	2.24 (1.64–3.06)	2.32E-05
rs2736114	T	0.27	0.26	0.97 (0.71–1.34)	0.8897
rs2075786	A	0.37	0.39	0.86 (0.65–1.14)	0.3756
rs4246742	A	0.15	0.17	0.87 (0.60–1.25)	0.5247
rs4975605	A	0.47	0.48	1.21 (0.91–1.60)	0.2666
rs10069690	T	0.27	0.27	0.97 (0.71–1.32)	0.859
rs2242652	A	0.19	0.20	1.27 (0.90–1.78)	0.261
rs2853677	G	0.45	0.42	0.84 (0.65–1.10)	0.2912
rs2853676	T	0.23	0.29	0.91 (0.68–1.22)	0.5973
rs2853672	C	0.51	0.46	0.75 (0.56–1.01)	0.1062

Model adjusted for maternal weight, education, income, smoking status, and insurance status.

SNP: single nucleotide polymorphism, MAF: minor allele frequency, PTL: preterm labor, aOR: adjusted odds ratio, CI: confidence interval

### Maternal SNP and pPROM association

The maternal SNP minor allele frequencies among 54 pPROM cases and 438 controls and the results of univariate analysis can be found in the supplement (S5 Table). Using the Bonferroni correction for multiple comparisons, the threshold for significance was set at p <0.005.

Of the 10 maternal SNPs analyzed, the same SNP that was associated with PTL, rs2853690, was also associated with pPROM on univariate comparison (OR 4.59, 95% CI 3.18–6.62, p = 3.0e-13), as well as one additional SNP, rs2736114 (OR 2.08, 95% CI 1.48–2.93, p = 0.0003) (S5 Table). Logistic regression analysis was also performed to assess the association with pPROM after adjusting for possible confounders (Table 5). Models adjusted for maternal age, education (graduate high school or not), income (annual <$15,000, $15,000–24,999, $25,000–49,999, $50,000–99,999, or ≥$100,000), marital status, smoker (Y/N), insurance status (Y/N), and sex of the infant. 32 cases and 368 controls were included in the logistic regression, after removing 22 cases and 70 controls with missing covariate data. Of the two SNPs that were significant on univariate analysis, only SNP rs2853690, the same one that was independently associated with PTL, remained significantly associated with pPROM (aOR 7.54, 95% CI 3.96–14.33, p = 2.39e-07)

**Table 5:** Logistic regression results for maternal single locus allele frequencies among cases and controls and association with preterm premature rupture of membranes.

SNP	Minor allele	MAF Term	MAF pPROM	aOR (95% CI)	P value
rs2853690	A	0.26	0.61	7.54 (3.96–14.33)	2.39E-07
rs2736114	T	0.27	0.44	2.16 (1.31–3.57)	0.01144
rs2075786	A	0.37	0.36	0.84 (0.53–1.35)	0.5541
rs4246742	A	0.15	0.14	0.58 (0.27–1.22)	0.2263
rs4975605	A	0.47	0.54	1.43 (0.90–2.28)	0.2038
rs10069690	T	0.27	0.22	1.09 (0.67–1.77)	0.7776
rs2242652	A	0.19	0.19	1.20 (0.68–2.12)	0.5968
rs2853677	G	0.45	0.33	0.66 (0.41–1.06)	0.1484
rs2853676	T	0.29	0.17	0.48 (0.27–0.87)	0.03968
rs2853672	C	0.51	0.36	0.50 (0.31–0.83)	0.02422

Model adjusted for maternal age, education, income, marital status, smoking status, insurance status, and sex of the infant.

SNP: single nucleotide polymorphism, MAF: minor allele frequency, pPROM: preterm premature rupture of membranes, aOR: adjusted odds ratio, CI: confidence interval

### Fetal SNP and PTL association

The fetal SNP minor allele frequencies among 116 PTL cases and 346 controls and the results of the univariate analysis are found in S3 Table. The Bonferroni correction yielded a threshold of p<0.006. Of the 8 fetal SNPs analyzed, no significant associations with PTL were found in either the adjusted (Table 6) or unadjusted models (S6 Table).

**Table 6:** Logistic regression results for fetal single locus allele frequencies among cases and controls and association with preterm labor.

SNP	Minor allele	MAF Term	MAF PTL	aOR (95% CI)	P
rs2736114	T	0.27	0.30	1.40 (0.99–1.98)	0.1057
rs2075786	A	0.42	0.42	0.85 (0.62–1.15)	0.374
rs4246742	A	0.16	0.15	0.72 (0.47–1.11)	0.2123
rs4975605	A	0.45	0.51	1.28 (0.95–1.72)	0.1757
rs10069690	T	0.27	0.28	1.07 (0.76–1.51)	0.7532
rs2242652	A	0.21	0.25	1.53 (1.04–2.24)	0.06831
rs2853677	G	0.44	0.49	1.05 (0.77–1.41)	0.81
rs2853672	C	0.48	0.48	0.83 (0.60–1.16)	0.3602

Model adjusted for maternal education, income, smoking status, and insurance status.

SNP: single nucleotide polymorphism, MAF: minor allele frequency, PTL: preterm labor, aOR: adjusted odds ratio, CI: confidence interval

### Fetal SNP and pPROM association

The fetal SNP minor allele frequencies among 40 pPROM cases and 346 controls and the results of the univariate analysis are found in S4 Table. The Bonferroni correction yielded a threshold of p<0.006. Of the 8 fetal SNPs analyzed, no significant associations with pPROM were found in either the adjusted (Table 7) or unadjusted models (S7 Table).

**Table 7:** Logistic regression results for fetal single locus allele frequencies among cases and controls and association with preterm premature rupture of membranes.

SNP	Minor allele	MAF Term	MAF pPROM	aOR (95% CI)	P
rs2736114	T	0.27	0.33	1.41 (0.85–2.37)	0.2611
rs2075786	A	0.42	0.38	0.69 (0.42–1.14)	0.2254
rs4246742	A	0.16	0.18	0.79 (0.42–1.51)	0.5523
rs4975605	A	0.45	0.56	1.44 (0.90–2.30)	0.1971
rs10069690	T	0.27	0.30	1.21 (0.72–2.03)	0.5508
rs2242652	A	0.21	0.24	1.19 (0.66–2.17)	0.6267
rs2853677	G	0.44	0.30	0.46 (0.26–0.78)	0.01734
rs2853672	C	0.48	0.32	0.45 (0.26–0.78)	0.01742

Model adjusted for maternal education, income, marital status, smoking status, insurance status, and sex of the infant.

SNP: single nucleotide polymorphism, MAF: minor allele frequency, pPROM: preterm premature rupture of membranes, aOR: adjusted odds ratio, CI: confidence interval

## Conclusion

Feto-maternal tissue senescence is a physiological factor associated with term parturition in humans. Premature and pathologic senescence activation in response to various pregnancy-associated risk factors can contribute to preterm labor and delivery.[25] Multitudes of risk factors have been linked to premature senescence activation; mainly oxidative stress inducing factors like infection/inflammation,[32–33] obesity,[34–35] nutritional factors,[36–37] and behavioral risks.[38–39] Oxidative stress induced by these risk factors can accelerate telomere attrition causing early aging and inflammation contributing to either PTL or pPROM. Genetic variation in maternal or fetal telomerase gene may also contribute to this process in the absence of specific risk factors, or an interaction between the two may also predispose to adverse pregnancy outcome. This study examined the existence of any genetic predisposition to PTL and/or pPROM through variations in the telomerase gene.

We report that *maternal* hTERT SNP rs2853690 was significantly associated with both PTL (aOR 2.24) and pPROM (aOR 7.54). Located in the non-coding region near the 3 prime end, the functional role of this SNP is unclear. This SNP is not in obvious linkage disequilibrium with any other variants in hTERT (r^2^ < 0.36 with all other SNPs in the gene) as determined from 1000 Genomes Project data. We found no significant associations between any of the analyzed *fetal* hTERT SNPs and PTL or pPROM.

The major strength of our study was that the samples came from an established biobank with well-defined phenotypes and well-characterized race and ethnicity. Quality control measures were used to ensure that only subjects with high quality genotyping and only SNPs with high genotyping efficiency were included. Additionally, we analyzed both maternal and newborn samples, allowing us to investigate both the maternal and fetal contributions to risk of preterm birth. Our candidate gene, hTERT, has a biologically plausible role in the mechanisms of PTL and pPROM. And finally, a significant strength of our study is that we adjusted for multiple comparisons using the Bonferroni correction, minimizing the risk that the association we found was due to type I error.

The study was limited by small sample sizes of cases, especially pPROM. Also, the findings may not be applicable to all populations because our study was restricted to Caucasians. Of interest, the risk allele (A) is at least twice as common in African ancestry populations as compared to European populations (https://www.ncbi.nlm.nih.gov/snp/), the former being at higher risk for PTL and pPROM).[40,41] However, seemingly inconsistent results are that germline telomere length appears to be longer in African individuals that those of European descent.[30] One explanation for this could be that specific genetic predispositions vary between races/ethnicities. A replication of our data in multiple independent cohorts, particularly in one that includes people of African descent, is essential prior to projecting the usefulness of this SNP as a genetic marker of high risk pregnancy. Another limitation of this study is that we have examined only SNPs in the hTERT gene and their association with PTB and pPROM. Other genetic and epigenetic variations in the hTERT gene may also contribute to functional alterations to hTERT activity or levels, contributing to adverse pregnancy outcomes.

Telomerase’s role in uterine and feto-maternal tissues is as area of active research. In women with recurrent implantation failure, expression of endometrial telomerase was enhanced during the implantation window.[42] In pregnancy, it has been shown that hTERT expression in the chorion is increased in the first trimester compared the second and third trimesters.[43] Similarly, telomerase activity level has been shown to be significantly higher in the first trimester in chorionic villi samples.[44] A study of human fetal tissues confirms the same pattern of decreasing telomere length and decreasing telomerase expression across the first trimester.[45]

Alterations in telomerase expression/activity have been shown to be associated with pathologic pregnancy states. While increased hTERT expression was noted in chorion cells of preeclamptic patients,[43] decreased telomerase activity has been observed in trophoblasts and placental biopsies from pregnancies affected by intrauterine growth restriction.[46–47] A study of growth discordant twins showed a tendency toward reduced telomerase activity in placental trophoblasts of the smaller twin.[48] While the results are not consistent, they may suggest a link between altered telomerase expression and/or activity and premature aging of fetal tissue leading to placental insufficiency and growth restriction.

In our continued work on telomerase’s role in preterm birth, our lab has subsequently performed hTERT gene activity and expression studies. We have found that telomerase has no activity in fetal membranes in the second and third trimesters, nor is it expressed in fetal membranes from either term or preterm placenta, including laboring and non-laboring samples. The lack of telomerase expression and activity in fetal membranes could explain why we see no association between infant genetic variation in hTERT and PTL or pPROM in this study. The lack of expression is also suggestive of unhindered telomere reduction to promote the natural progression of senescence that will eventually result in parturition. This study identifies a biologically plausible candidate gene for replicative studies and contributes to the growing evidence that replicative senescence plays a role in maternal-fetal signaling of parturition.

## Supporting information

S1 Table Genotyping and covariate data. MB: maternal blood, CB: cord blood, FamID: family identification number, IndID: individual identification number, GES_WGHT: birth weight.
(XLSX)

S2 Table 1 and 5 minute APGAR scores of neonates of controls and cases in the two maternal analyses. PTL: preterm labor, pPROM: preterm premature rupture of membranes.
(DOCX)

S3 Table 1 and 5 minute APGAR scores of neonates of controls and cases in the two fetal analyses. PTL: preterm labor, pPROM: preterm premature rupture of membranes.
(DOCX)

S4 Table Maternal single locus allele frequencies among cases and controls and association with preterm labor (unadjusted model). SNP: single nucleotide polymorphism, MAF: minor allele frequency, PTL: preterm birth, OR: odds ratio, CI: confidence interval.
(DOCX)

S5 Table Maternal single locus allele frequencies among cases and controls and association with preterm premature rupture of membranes (unadjusted model). SNP: single nucleotide polymorphism, MAF: minor allele frequency, pPROM: preterm premature rupture of membranes, OR: odds ratio, CI: confidence interval.
(DOCX)

S6 Table Fetal single locus allele frequencies among cases and controls and association with preterm labor (unadjusted model). SNP: single nucleotide polymorphism, MAF: minor allele frequency, PTL: preterm labor, OR: odds ratio, CI: confidence interval.
(DOCX)

S7 Table Fetal single locus allele frequencies among cases and controls and association with preterm premature rupture of membranes. SNP: single nucleotide polymorphism, MAF: minor allele frequency, pPROM: preterm premature rupture of membranes, OR: odds ratio, CI: confidence interval.
(DOCX)
