# Supplementary material for: Maternal human telomerase reverse transcriptase variants are associated with preterm labor and preterm premature rupture of membranes
Source: PLoS One. 2018 May 17;13(5):e0195963. doi: 10.1371/journal.pone.0195963 (PMC5957404; doi:10.1371/journal.pone.0195963)
Supplement: S2 Table — PTL: preterm labor, pPROM: preterm premature rupture of membranes. (DOCX) [file pone.0195963.s002.docx]

**Supporting information**

S2 Table. 1 and 5 minute APGAR scores of neonates of controls and cases in the two maternal analyses.

|  | |  | **Maternal PTL** | | | **Maternal pPROM** | | |
| --- | --- | --- | --- | --- | --- | --- | --- | --- |
| **Score** | |  | **Control N=438** | **Case N=162** | **P value** | **Control N=438** | **Case N=54** | **P value** |
| APGAR1 |  | 1 | 0.004 | 0.01 | 1E-12 | 0.004 | 0.061 | 1E-15 |
|  |  | 2 | 0.004 | 0.013 |  | 0.004 | 0 |  |
|  |  | 3 | 0.004 | 0.006 |  | 0.004 | 0 |  |
|  |  | 4 | 0.009 | 0.051 |  | 0.009 | 0 |  |
|  |  | 5 | 0.002 | 0.025 |  | 0.002 | 0.061 |  |
|  |  | 6 | 0.011 | 0.089 |  | 0.011 | 0.041 |  |
|  |  | 7 | 0.064 | 0.14 |  | 0.064 | 0.245 |  |
|  |  | 8 | 0.437 | 0.465 |  | 0.437 | 0.388 |  |
|  |  | 9 | 0.464 | 0.197 |  | 0.464 | 0.184 |  |
|  |  | 10 | 0 | 0 |  | 0 | 0.02 |  |
| APGAR5 |  | 1 | 0.004 | 0 | 1E-18 | 0.004 | 0.041 | 3E-16 |
|  |  | 2 | 0 | 0 |  | 0 | 0 |  |
|  |  | 3 | 0 | 0 |  | 0 | 0 |  |
|  |  | 4 | 0 | 0 |  | 0 | 0.02 |  |
|  |  | 5 | 0 | 0.006 |  | 0 | 0 |  |
|  |  | 6 | 0.004 | 0.032 |  | 0.004 | 0.02 |  |
|  |  | 7 | 0.009 | 0.051 |  | 0.009 | 0.082 |  |
|  |  | 8 | 0.033 | 0.223 |  | 0.033 | 0.245 |  |
|  |  | 9 | 0.934 | 0.675 |  | 0.934 | 0.571 |  |
|  |  | 10 | 0.015 | 0.013 |  | 0.015 | 0.02 |  |

PTL: preterm labor, pPROM: preterm premature rupture of membranes
