# Supplementary material for: Maternal human telomerase reverse transcriptase variants are associated with preterm labor and preterm premature rupture of membranes
Source: PLoS One. 2018 May 17;13(5):e0195963. doi: 10.1371/journal.pone.0195963 (PMC5957404; doi:10.1371/journal.pone.0195963)
Supplement: S3 Table — PTL: preterm labor, pPROM: preterm premature rupture of membranes. (DOCX) [file pone.0195963.s003.docx]

**Supporting information**

S3 Table. 1 and 5 minute APGAR scores of neonates of controls and cases in the two fetal analyses.

|  | |  | **Fetal PTL** | | | **Fetal pPROM** | | |
| --- | --- | --- | --- | --- | --- | --- | --- | --- |
| **Score** | |  | **Control N=438** | **Case N=162** | **P value** | **Control N=438** | **Case N=40** | **P value** |
| APGAR1 |  | 1 | 0 | 0.009 | 2E-09 | 0 | 0 | 0.000213 |
|  |  | 2 | 0.006 | 0.026 |  | 0.006 | 0.051 |  |
|  |  | 3 | 0 | 0.017 |  | 0 | 0 |  |
|  |  | 4 | 0.003 | 0.026 |  | 0.003 | 0 |  |
|  |  | 5 | 0.003 | 0.026 |  | 0.003 | 0.026 |  |
|  |  | 6 | 0.014 | 0.06 |  | 0.014 | 0 |  |
|  |  | 7 | 0.048 | 0.146 |  | 0.048 | 0.154 |  |
|  |  | 8 | 0.459 | 0.483 |  | 0.459 | 0.41 |  |
|  |  | 9 | 0.467 | 0.207 |  | 0.467 | 0.359 |  |
|  |  | 10 | 0 | 0 |  | 0 | 0 |  |
| APGAR5 |  | 1 | 0.003 | 0 | 7E-12 | 0.003 | 0 | 0.00002 |
|  |  | 2 | 0 | 0 |  | 0 | 0 |  |
|  |  | 3 | 0 | 0 |  | 0 | 0 |  |
|  |  | 4 | 0 | 0.009 |  | 0 | 0 |  |
|  |  | 5 | 0 | 0.009 |  | 0 | 0 |  |
|  |  | 6 | 0 | 0.017 |  | 0 | 0.051 |  |
|  |  | 7 | 0.011 | 0.052 |  | 0.011 | 0.026 |  |
|  |  | 8 | 0.04 | 0.241 |  | 0.04 | 0.103 |  |
|  |  | 9 | 0.929 | 0.655 |  | 0.929 | 0.795 |  |
|  |  | 10 | 0.017 | 0.017 |  | 0.017 | 0.026 |  |

PTL: preterm labor, pPROM: preterm premature rupture of membranes
