# Supplementary material for: Maternal human telomerase reverse transcriptase variants are associated with preterm labor and preterm premature rupture of membranes
Source: PLoS One. 2018 May 17;13(5):e0195963. doi: 10.1371/journal.pone.0195963 (PMC5957404; doi:10.1371/journal.pone.0195963)
Supplement: S4 Table — SNP: single nucleotide polymorphism, MAF: minor allele frequency, PTL: preterm birth, OR: odds ratio, CI: confidence interval. (DOCX) [file pone.0195963.s004.docx]

**Supporting information**

S4 Table. Maternal single locus allele frequencies among cases and controls and association with preterm labor (unadjusted model)

| **SNP** | **Minor allele** | **MAF Term** | **MAF PTL** | **OR (95% CI)** | **P value** |
| --- | --- | --- | --- | --- | --- |
| rs2853690 | A | 0.26 | 0.46 | 2.42 (1.93-3.05) | 8E-11 |
| rs2736114 | T | 0.27 | 0.26 | 0.96 (0.75-1.22) | 0.78 |
| rs2075786 | A | 0.37 | 0.39 | 1.11 (0.89-1.39) | 0.43 |
| rs4246742 | A | 0.15 | 0.17 | 1.18 (0.89-1.56) | 0.35 |
| rs4975605 | A | 0.47 | 0.48 | 1.06 (0.85-1.31) | 0.67 |
| rs10069690 | T | 0.27 | 0.27 | 1.00 (0.79-1.27) | 0.99 |
| rs2242652 | A | 0.19 | 0.20 | 1.03 (0.78-1.35) | 0.88 |
| rs2853677 | G | 0.45 | 0.42 | 0.91 (0.73-1.13) | 0.47 |
| rs2853676 | T | 0.29 | 0.29 | 0.99 (0.78-1.25) | 0.95 |
| rs2853672 | C | 0.51 | 0.46 | 0.84 (0.68-1.05) | 0.22 |

SNP: single nucleotide polymorphism, MAF: minor allele frequency, PTL: preterm birth, OR: odds ratio, CI: confidence interval
