# Supplementary material for: Maternal human telomerase reverse transcriptase variants are associated with preterm labor and preterm premature rupture of membranes
Source: PLoS One. 2018 May 17;13(5):e0195963. doi: 10.1371/journal.pone.0195963 (PMC5957404; doi:10.1371/journal.pone.0195963)
Supplement: S5 Table — SNP: single nucleotide polymorphism, MAF: minor allele frequency, pPROM: preterm premature rupture of membranes, OR: odds ratio, CI: confidence interval. (DOCX) [file pone.0195963.s005.docx]

**Supporting information**

S5 Table. Maternal single locus allele frequencies among cases and controls and association with preterm premature rupture of membranes (unadjusted model)

| **SNP** | **Minor allele** | **MAF Term** | **MAF pPROM** | **OR (95% CI)** | **P value** |
| --- | --- | --- | --- | --- | --- |
| rs2853690 | A | 0.26 | 0.61 | 4.59 (3.18-6.62) | 3E-13 |
| rs2736114 | T | 0.27 | 0.44 | 2.08 (1.48-2.93) | 3E-4 |
| rs2075786 | A | 0.37 | 0.36 | 0.84 (0.53-1.35) | 0.82 |
| rs4246742 | A | 0.15 | 0.14 | 0.58 (0.27-1.22) | 0.75 |
| rs4975605 | A | 0.47 | 0.54 | 1.43 (0.90-2.28) | 0.19 |
| rs10069690 | T | 0.27 | 0.22 | 1.09 (0.67-1.77) | 0.29 |
| rs2242652 | A | 0.19 | 0.19 | 1.20 (0.68-2.12) | 0.84 |
| rs2853677 | G | 0.45 | 0.33 | 0.66 (0.41-1.06) | 0.02 |
| rs2853676 | T | 0.29 | 0.17 | 0.48 (0.27-0.86) | 0.007 |
| rs2853672 | C | 0.51 | 0.36 | 0.50 (0.31-0.83) | 0.0059 |

SNP: single nucleotide polymorphism, MAF: minor allele frequency, pPROM: preterm premature rupture of membranes, OR: odds ratio, CI: confidence interval
