# Supplementary material for: Maternal human telomerase reverse transcriptase variants are associated with preterm labor and preterm premature rupture of membranes
Source: PLoS One. 2018 May 17;13(5):e0195963. doi: 10.1371/journal.pone.0195963 (PMC5957404; doi:10.1371/journal.pone.0195963)
Supplement: S6 Table — SNP: single nucleotide polymorphism, MAF: minor allele frequency, PTL: preterm labor, OR: odds ratio, CI: confidence interval. (DOCX) [file pone.0195963.s006.docx]

**Supporting information**

S6 Table. Fetal single locus allele frequencies among cases and controls and association with preterm labor (unadjusted model)

| **SNP** | **Minor allele** | **MAF Term** | **MAF PTL** | **OR (95% CI)** | **P value** |
| --- | --- | --- | --- | --- | --- |
| rs2736114 | T | 0.34 | 0.30 | 1.40 (0.99-1.98) | 0.32 |
| rs2075786 | A | 0.56 | 0.42 | 0.86 (0.62-1.15) | 0.79 |
| rs4246742 | A | 0.19 | 0.15 | 0.72 (0.47-1.11) | 0.40 |
| rs4975605 | A | 0.59 | 0.51 | 1.28 (0.95-1.72) | 0.10 |
| rs10069690 | T | 0.34 | 0.28 | 1.07 (0.76-1.51) | 0.91 |
| rs2242652 | A | 0.25 | 0.25 | 1.53 (1.04-2.24) | 0.26 |
| rs2853677 | G | 0.56 | 0.49 | 1.05 (0.77-1.41) | 0.38 |
| rs2853672 | C | 0.64 | 0.48 | 0.83 (0.60-1.16) | 0.77 |

SNP: single nucleotide polymorphism, MAF: minor allele frequency, PTL: preterm labor, OR: odds ratio, CI: confidence interval
