# Supplementary material for: Maternal human telomerase reverse transcriptase variants are associated with preterm labor and preterm premature rupture of membranes
Source: PLoS One. 2018 May 17;13(5):e0195963. doi: 10.1371/journal.pone.0195963 (PMC5957404; doi:10.1371/journal.pone.0195963)
Supplement: S7 Table — SNP: single nucleotide polymorphism, MAF: minor allele frequency, pPROM: preterm premature rupture of membranes, OR: odds ratio, CI: confidence interval. (DOCX) [file pone.0195963.s007.docx]

**Supporting information**

S7 Table. Fetal single locus allele frequencies among cases and controls and association with preterm premature rupture of membranes

| **SNP** | **Minor allele** | **MAF Term** | **MAF pPROM** | **OR (95% CI)** | **P value** |
| --- | --- | --- | --- | --- | --- |
| rs2736114 | T | 0.34 | 0.33 | 1.42 (0.85-2.37) | 0.18 |
| rs2075786 | A | 0.56 | 0.38 | 0.69 (0.42-1.14) | 0.38 |
| rs4246742 | A | 0.19 | 0.18 | 0.79 (0.42-1.51) | 0.67 |
| rs4975605 | A | 0.59 | 0.56 | 1.44 (0.90-2.30) | 0.13 |
| rs10069690 | T | 0.34 | 0.29 | 1.21 (0.72-2.03) | 0.56 |
| rs2242652 | A | 0.25 | 0.24 | 1.19 (0.66-2.17) | 0.55 |
| rs2853677 | G | 0.56 | 0.30 | 0.46 (0.26-0.78) | 0.03 |
| rs2853672 | C | 0.64 | 0.32 | 0.45 (0.26-0.78) | 0.02 |

SNP: single nucleotide polymorphism, MAF: minor allele frequency, pPROM: preterm premature rupture of membranes, OR: odds ratio, CI: confidence interval
